# An Examination of Women's Self-Presentation, Social Physique Anxiety, and Setting Preferences during Injury Rehabilitation

**DOI:** 10.1155/2017/6126509

**Published:** 2017-03-12

**Authors:** Molly V. Driediger, Carly D. McKay, Craig R. Hall

**Affiliations:** ^1^Western University, London, ON, Canada; ^2^Centre for Motivation and Health Behaviour Change, Department for Health, University of Bath, Bath, UK

## Abstract

*Objectives*. This study investigated whether women experience self-presentational concerns related to rehabilitation settings and explored preferences for characteristics of the social and physical treatment environment in relation to women's Social Physique Anxiety (SPA).* Methods*. Two cross-sectional studies were conducted. In Study 1, female undergraduate students (*n* = 134) completed four questionnaires (Social Physique Anxiety Scale; three bespoke questionnaires assessing self-presentation in rehabilitation and social and physical environment preferences) with respect to hypothetical rehabilitation scenarios. Study 2 recruited injured women who were referred for physiotherapy (*n* = 62) to complete the same questionnaires regarding genuine rehabilitation scenarios.* Results*. Women with high SPA showed less preference for physique salient clothing than women with low SPA in both hypothetical (*p* = 0.001) and genuine settings (*p* = 0.01). In Study 2, women with high SPA also preferred that others in the clinic were female (*p* = 0.01) and reported significantly greater preference for private treatment spaces (*p* = 0.05).* Conclusions*. Self-presentational concerns exist in rehabilitation as in exercise settings. Results indicated inverse relationships between women's SPA and preference for the presence of men, physique-enhancing clothing, and open-concept treatment settings. Future studies to determine the effect of self-presentational concerns on treatment adherence are needed.

## 1. Introduction

Self-presentation is the effort people make to control how they are perceived by others [1, 2]. People differ in their degree of self-presentation and may experience social anxiety when the impressions they wish to convey are incongruent with their perceived ability to present those impressions [3]. Social anxiety related to the evaluation of one's physique is termed Social Physique Anxiety (SPA) [4]. SPA has been explored extensively in exercise and is emerging as a salient concern in injury rehabilitation [5, 6].

Women's self-presentational concerns consistently exceed those expressed by men [4, 7–10] and those high in trait SPA are particularly sensitive to the social and physical elements of the environment [11–13]. For example, the presence of men in exercise settings has been shown to significantly increase women's state levels of SPA [9, 14]. Women possessing high dispositional SPA typically prefer exercising with exclusively female groups [9, 15] and often shorten their workouts when men are present [9]. The physique and physical ability of others have also been shown to influence anxiety, such that women with greater SPA prefer not to exercise with others who are more fit than they are [12, 16–18]. There is evidence that wearing clothing that emphasizes the body also results in elevated SPA [11–13, 18] and women with high trait SPA prefer wearing clothing that deemphasizes their physique [11–13, 19].

Elements of the physical environment can also influence self-presentation. In general, women with high SPA tend to choose exercise locations with less chance of physical evaluation, preferring to exercise in private rather than in public [20]. The presence of mirrors and windows has been shown to heighten women's awareness of their physique, consequently increasing SPA [13, 21].

Research in exercise has linked self-presentational concerns to low adherence rates and indicates that SPA may discourage people from exercising altogether, especially when the environment is viewed as threatening [3, 22]. The social and physical elements of exercise settings are similar to those found in clinical rehabilitation environments, and it is likely that negative behavioural outcomes may also occur in this context [23]. Yet, limited research to-date has explicitly examined self-presentation in injury rehabilitation [5, 6]. Thus, the objective of the present research was to determine whether women's self-presentational concerns in rehabilitation are similar to those found in exercise settings.

To address this objective, two studies were conducted. The purpose of Study 1 was to demonstrate proof-of-concept by investigating whether healthy women experience self-presentational concerns related to hypothetical rehabilitation settings. Study 2 extended these findings by exploring the self-presentational concerns of injured women beginning a rehabilitation program. For both studies, it was hypothesized that women with high SPA would prefer same-sex rehabilitation environments, clothing that deemphasized their physique, and private treatment settings. It was also expected that there would be a positive correlation between SPA and preferences to be treated by a female physiotherapist.

## 2. Study 1

### 2.1. Methods

#### 2.1.1. Design and Participants

This cross-sectional study included a convenience sample of female students recruited from three undergraduate classes at a Canadian university. Investigators approached each class at the beginning of a lecture and provided questionnaire packages to consenting students. Individuals currently undergoing physiotherapy were ineligible to participate, and responses from men were excluded from the analysis. Approval for this study was obtained from the Western University ethics board.

#### 2.1.2. Outcome Measures

Participants were asked to provide detailed demographic information including age, height, and weight. They were also asked to complete a package of four self-report questionnaires.

*Social Physique Anxiety Scale (SPAS).* This 9-item scale, which has been found to be psychometrically sound [24], measures concern with the evaluation of one's figure or physique [4]. Participants rate each statement on a 5-point scale ranging from 1 (not at all characteristic of me) to 5 (extremely characteristic of me). 

*Self-Presentation in Injury Rehabilitation Questionnaire (SPIRQ).* This 32-item questionnaire was developed for the current study to assess self-presentational concerns in physiotherapy (see supplementary content in Supplementary Material available online at https://doi.org/10.1155/2017/6126509). Items were generated from an examination of the literature and two existing measures of self-presentation in sport and exercise: the Self-Presentation in Sport Questionnaire (SPSQ) [25] and the Self-Presentation in Exercise Questionnaire (SPEQ) [26]. Participants were asked to imagine that they were undergoing rehabilitation for a specified injury. A written description of a physiotherapy clinic was provided, along with photographs depicting the physical features of the clinic environment and information about the people who would be present during the hypothetical treatment. Each questionnaire item began with the stem: “I believe the other people in the clinic would perceive me as…” and listed a number of items (e.g., self-conscious, uncoordinated, and unable to perform exercises). Participants were instructed to rate each item on a scale ranging from 1 (not at all true) to 5 (completely true). 

*Injury Rehabilitation Social Environment Preferences Questionnaire*. This 14-item questionnaire was developed for the current study to evaluate preferences for aspects of the social environment of a physiotherapy clinic (see supplementary online content). The items consisted primarily of characteristics representing the other people present in the clinic (i.e., sex [*n* = 3], physique/physical ability [*n* = 2], level of interaction [*n* = 2], need to impress [*n* = 2], and sex of therapist [*n* = 2]) as well as three items that referred to the clothing that participants may be required to wear. Participants rated their preference for each item on a 5-point scale with the anchors* not preferred *(1),* no preference *(3), and* completely prefer *(5). 

*Injury Rehabilitation Treatment Environment Preferences Questionnaire*. This 3-item questionnaire was also developed for the present study to assess preferences for specific treatment settings found within physiotherapy clinics (see supplementary online content). Items included a written description of the physical treatment environment with a photograph to augment the description. The design features and treatment descriptions illustrated three different degrees of potential for evaluation from others in the clinic (e.g., high, medium, and low). Participants were asked to rate each item based on 5 points with the anchors* not preferred* (1),* no preference* (3), and* completely prefer* (5).

#### 2.1.3. Analysis

Bivariate correlations were calculated for SPAS total score and each of the 32 SPIRQ items. SPIRQ items were analyzed individually as this questionnaire does not represent a scale.

Bivariate correlations were calculated to evaluate the relationship between SPA and rehabilitation environment preferences. Additionally, in order to distinguish between those who were highly physique anxious and those who experienced low levels of SPA, participants were divided based on SPA score into low (10–20), medium (21–28), and high (29–41) groups using an approximate tertile split. The extreme group approach (EGA) has been employed previously in the sport psychology literature [27] and although criticized as it amplifies power and effect size, the use of EGA may be beneficial if the study is exploratory in nature and aims to establish the existence and direction of an effect, but not its strength [28]. Thus, an ANOVA was performed with SPA [low (*n* = 43), moderate (*n* = 47), and high (*n* = 44)] as the independent variable and the social and physical preference items as the dependent variables. Tukey's HSD post hoc tests were performed to determine differences between the three levels of SPA.

For all analyses, a less conservative alpha of *p* ≤ 0.05 was employed because of the exploratory nature of the study. No correction was made for multiple comparisons.

### 2.2. Results

Of 193 students approached, three chose not to participate and two were omitted because of incomplete data. Fifty-four men were excluded, resulting in a final sample of 134 women. Participant characteristics are presented in Table 1. In this sample, 58.2% of women had previously experienced physiotherapy treatment.

#### 2.2.1. Self-Presentational Concerns and SPA

Twenty of the 32 SPIRQ items were significantly related to SPA. Pearson *r* values ranged from −1.00 to 0.47 (Table 2).

#### 2.2.2. Injury Rehabilitation Environment Preferences

ANOVA found a significant effect for wearing nonphysique salient clothing (i.e., loose fitting long pants and a long-sleeved shirt) [*F*(2,133) = 7.57, *p* = 0.00, *η*^2^ = 0.10]. Post hoc tests revealed significant differences between low SPA and high SPA groups (*p* = 0.001), as well as low SPA and moderate SPA groups (*p* = 0.01). Thus, compared to those with lower SPA, women with higher SPA indicated significantly greater preference for wearing nonphysique salient clothing. Alternatively, women with low SPA preferred dressing in physique-enhancing clothing (i.e., short, tight spandex top, and bottoms) more than their high SPA scoring counterparts [*F*(2,133) = 5.38, *p* = 0.01, *η*^2^ = 0.08], as post hoc tests showed significant differences only between women who scored low and high on SPA (*p* = 0.001). Additionally, there was a significant effect for open-concept treatment settings [*F*(2,133) = 3.59, *p* = 0.03, *η*^2^ = 0.05]. Significant differences were found between low SPA and high SPA women (*p* = 0.02) indicating women with low SPA preferred open-concept treatment settings compared to high SPA women. No other differences between high and low SPA participants emerged (Table 3).

### 2.3. Discussion

The findings indicate that healthy women exhibit self-presentational concerns when presented with hypothetical rehabilitation scenarios. As hypothesized, the results demonstrate that preferences for treatment environments are associated with SPA, which is consistent with the exercise literature [9, 12, 13, 18].

Unexpectedly, the gender of the physiotherapist was not related to SPA. There is considerable evidence; however, that interpersonal characteristics may override concerns over practitioner gender in treatment settings [29]. Further investigation into the role of the physiotherapist in promoting or mitigating social anxiety is thus warranted to better understand this relationship.

As hypothesized, as SPA increased, women demonstrated less preference for clothing that emphasized their physique and greater preference for wearing clothing that camouflaged their body type. This is consistent with previous studies in the exercise domain [11–13]. Also, as SPA increased, women reported less preference for being treated in an open-concept environment, but no significant relationship was found between SPA and a preference for being treated behind a closed door. This finding was unexpected, given that an examination room would offer the most privacy, and previous research has demonstrated that women with high SPA choose exercise settings that are private [5, 20]. The lack of expressed preference for the most private setting may have been a reflection of the current sample of primarily young, average weight, healthy women with moderate SPA. For example, injured women with high SPA might respond differently when faced with the reality of a clinical environment [5] Furthermore, the current sample of students may be desensitized to the evaluative potential of various settings because of the type of educational experience they have had, and the majority of our sample reported previous physiotherapy experience [12]. This exposure may have acted to reduce reported anxiety related to the physical rehabilitation environment.

Study 1 demonstrated that self-presentational concerns based on hypothetical scenarios are similar to those existing in the exercise literature. In Study 2 we sought to replicate and extend our findings in a sample of injured women who were initiating physiotherapy.

## 3. Study 2

### 3.1. Methods

#### 3.1.1. Design and Participants

In this cross-sectional study, participants were injured women (*n* = 62) who were referred for physiotherapy. Potential participants were identified and approached by their primary care practitioner at clinics in two Canadian cities. All women who were referred for physiotherapy during the study period were screened for eligibility. Women were ineligible to participate if they did not speak or read English or had already begun physiotherapy treatment. Consenting participants completed a questionnaire package prior to their initial physiotherapy appointment. Ethical approval was obtained from the Western University ethics board.

#### 3.1.2. Measures

Women were asked to provide detailed demographic information and completed the SPAS, SPIRQ, Injury Rehabilitation Social Environment Preferences questionnaire, and Injury Rehabilitation Treatment Environment Preferences questionnaire (as in Study 1).

#### 3.1.3. Analysis

Seven cases had multiple missing questionnaire responses but were retained due to the small sample size. Missing values (*n* < 10%) were replaced by the series mean.

Bivariate correlations were calculated for SPAS total score and each of the 32 SPIRQ items. Bivariate correlations were also calculated to evaluate the relationship between SPA and rehabilitation environment preferences. As in Study 1, participants were divided based on SPA score into low (11–22), medium (23–27), and high (28–45) groups using an approximate tertile split. An ANOVA was performed with SPA (low [*n* = 21], medium [*n* = 20], and high [*n* = 21]) as the independent variable and the social and physical preference items as the dependent variables. Tukey HSD post hoc tests were performed to determine differences between SPA groups.

Again, a less conservative alpha of *p* ≤ 0.05 was employed because of the exploratory nature of the study and there was no correction for multiple comparisons.

### 3.2. Results

Two patients were excluded based on English proficiency (*n* = 2), and one because she had already begun treatment. One patient chose not to participate. The characteristics of participants are presented in Table 4. The women in this sample (*n* = 62) reported relatively high SPA (M = 25.44, SD = 7.54), and as BMI increased, so did SPA (Pearson *r* = 0.29).

#### 3.2.1. Self-Presentational Concerns

There was a significant, positive relationship between SPA and eight of the 32 items of the SPIRQ (Table 5). Correlated items represented the psychological and physical manifestations of anxiety (e.g., anxious, self-conscious, and tense), as well as negative descriptions of physique (e.g., unfit, unhealthy).

#### 3.2.2. Injury Rehabilitation Environment Preferences

ANOVA demonstrated a significant effect for a preference for attending physiotherapy in an exclusively female setting [*F*(2,61) = 4.58, *p* = 0.01, *η*^2^ = 0.13]. Post hoc tests revealed significant differences (*p* = 0.01) between women who reported moderate levels of SPA and women who had high levels of SPA. No significant differences were found between women with low SPA and women with high SPA.

There was also a significant preference for wearing short, tight fitting clothing [*F*(2,61) = 5.42, *p* = 0.01, *η*^2^ = 0.16]. Further analysis indicated that compared to women who reported moderate and high SPA, women who indicated low SPA revealed greater preference for wearing short, tight fitting clothing during treatment (*p* = 0.01). A significant preference existed for receiving treatment in an open-concept treatment space [*F*(2,61) = 4.37, *p* = 0.02, *η*^2^ = 0.13]. Post hoc tests revealed that low SPA scoring women showed a significantly greater preference for receiving treatment in an open space compared to women with moderate and high SPA (*p* = 0.03 and *p* = 0.04, resp.). Finally, there was a significant effect for being treated in a private room [*F*(2,61) = 3.14, *p* = 0.05, *η*^2^ = 0.1]. Women with high SPA preferred being treated in a private exam room compared to women who scored low on SPA as post hoc tests showed significant differences between low SPA and high SPA groups (*p* = 0.05) (Table 6).

### 3.3. Discussion

Consistent with Study 1, positive relationships were revealed between SPA and appearance/physical fitness descriptors. Given the distinct samples in Study 1 and Study 2, this consistency implies that there may be some persistent self-presentational concerns for women regardless of age, fitness, or SPA. However, fewer items from the SPIRQ were found to relate significantly to SPA in the present study (8 items) compared to Study 1 (20 items). Women in the current study were actually initiating a physiotherapy program, however, and were recruited from community-based clinics. The results of Study 2, therefore, likely provide a more accurate portrayal of the salient self-presentational concerns in general rehabilitation settings.

As hypothesized, yet unlike the findings of Study 1, women with the highest levels of SPA reported greater preference for receiving treatment alongside other patients who were female [9, 12]. Surprisingly, this preference was not found to differ significantly between women who experienced the lowest levels of SPA and those with high SPA, suggesting that there may be additional factors that shape social environment preferences in this population which bear further investigation. Importantly, in an examination of injured women who exhibited high levels of SPA (>25 on SPAS), the presence of men was specified as a barrier to women's attendance at physiotherapy sessions [5]. There is also evidence that the presence of men may act to reduce the duration of women's exercise sessions or limit the enjoyment they derive from exercise [9, 13]. Thus, our findings imply that a male presence may influence some women's rehabilitation behaviour. Future studies should therefore examine how treatment adherence is affected by the relationship between SPA and the presence of men in physiotherapy clinics. Interestingly, participants again did not express a preference for their physiotherapist to be female. This further suggests that the knowledge, experience, and confidence that a physiotherapist demonstrates are indicative of effective treatment and may be more important to patients than self-presentational concerns [29].

In keeping with Study 1, a significant relationship between level of SPA and clothing choice emerged. That is, women who were highly physique anxious indicated a lesser preference for wearing clothing that accentuated their body. Similarly, in exercise settings, women who are high in dispositional SPA prefer clothing that deemphasizes their figure [11–13, 19]. When physical elements of the rehabilitation clinic were examined, injured women who scored highest in SPA preferred treatment environments that provided privacy, specifically behind closed doors. The expressed preference for private examination rooms was inconsistent with the results of Study 1, yet supported our hypotheses, and likely provided a more accurate representation of the treatment setting preferences of women who are actually undergoing injury rehabilitation. Consistent with Study 1, women low in physique anxiety showed greater preference for open-concept treatment settings compared to those who were high in SPA. These findings highlight the distinct treatment setting preferences of women who experience different levels of physique anxiety in relation to injury rehabilitation.

## 4. Summary and Conclusions

Several limitations to this research must be acknowledged. The questionnaires used to measure self-presentation were exploratory in nature, and the validity and reliability of these measures have not been well established. Our studies were also underpowered and as such may have led to an underestimation of the strength of the associations between self-presentational concerns and environmental preferences. As this research was exploratory in nature, we took a descriptive approach in order to provide a basic understanding of women's self-presentational concerns in rehabilitation. These results therefore should not be used to inform practice but are intended to support future hypothesis generation, study design, and sample size calculation.

Despite the limitations, this research provides evidence that self-presentational concerns exist in the context of injury rehabilitation. In particular, we demonstrated inverse relationships between women's SPA and preference for the presence of men, clothing that accentuates the physique, and open-concept treatment settings. Self-presentational concerns can negatively impact exercise adherence, and it is likely that the same is true for rehabilitation [3, 22]. Therefore, it will be important to determine how elevated SPA may influence women's rehabilitation behaviour, particularly with respect to treatment adherence.

## Supplementary Material

The Supplementary Material contains the bespoke questionnaires created for the reported studies: the Self-Presentation in Injury Rehabilitation Questionnaire (SPIRQ), the Injury Rehabilitation Social Environment Preferences Questionnaire, and the Injury Rehabilitation Treatment Environment Preferences Questionnaire.

## Figures and Tables

**Table 1:** Descriptive characteristics of students included in Study 1.

Characteristic	Women (*n* = 134)
	Median (range)
Age	20 (18–39)
Height (m)	1.68 (1.52–1.96)
Weight (kg)	61.24 (47.63–96.16)
BMI (kg/m^2^)	21.93 (16.01–30.42)
SPA	25.00 (10.00–41.00)

*Note*. BMI = Body Mass Index; SPA = Social Physique Anxiety.

**Table 2:** Ratings for SPIRQ items and correlation with SPA for female students.

Item	Women (*n* = 134)		
	Mean	SD	Pearson *r*
Weak	1.66	0.90	0.19^*∗*^
Anxious	1.85	0.99	0.29^*∗∗*^
Self-conscious	2.13	1.08	0.46^*∗∗*^
Relaxed	2.53	1.09	0.33^*∗∗*^
Uncoordinated	1.80	0.96	0.15
Unable to perform	1.66	0.83	0.17^*∗*^
Healthy	2.16	0.86	0.33^*∗∗*^
Fit	2.33	0.91	0.42^*∗∗*^
Unfocused	1.67	0.84	0.05
Energized	2.57	0.96	0.28^*∗∗*^
Difficult to work with	1.22	0.62	0.20^*∗*^
Tense	2.28	1.06	0.20^*∗*^
Strong	2.79	0.98	0.16
Composed	2.41	0.77	0.17^*∗*^
Unable to handle pressure	1.61	0.87	0.19^*∗*^
Frustrated	2.05	1.01	0.21^*∗*^
Unconcerned	2.16	1.02	−1.00
Coordinated	2.46	0.94	0.06
Unfit	1.69	0.96	0.46^*∗∗*^
In control of emotions	2.03	0.86	0.11
Focused	2.08	0.77	0.20^*∗*^
Tired	2.29	0.96	0.04
Confident	2.46	0.90	0.42^*∗∗*^
Dumb	1.23	0.68	0.16
Unhealthy	1.46	0.77	0.47^*∗∗*^
Easy to work with	1.78	0.82	0.15
Knowledgeable	2.15	0.73	0.15
Able to handle pressure	2.19	0.85	0.17
Unable to control emotions	1.53	0.94	0.01
Needy	1.55	0.92	0.21^*∗*^
Perform exercises well	2.17	0.74	0.26^*∗∗*^
Independent	2.05	0.87	0.32^*∗∗*^

*Note*. Pearson *r* values depict the correlation with SPA. SPA = Social Physique Anxiety. ^*∗*^*p* < 0.05, ^*∗∗*^*p* < 0.01.

**Table 3:** Environmental preferences for female students.

Item	Women (*N* = 134)	Low SPA (*n* = 43)	Med SPA (*n* = 47)	High SPA (*n* = 44)	Pearson *r* (total sample)
	Mean (SD)				
*Social environment*					
The other patients in the clinic are female	3.37 (0.75)	3.25 (0.62)	3.30 (0.83)	3.57 (0.77)	0.18^*∗*^
The other patients in the clinic are male	2.66 (0.85)	2.95 (0.83)	2.57 (0.77)	2.44 (0.88)	−0.27^*∗∗∗*^
There are an equal number of males and females in the clinic	3.07 (0.67)	3.16 (0.65)	3.11 (0.76)	2.93 (0.59)	−0.17^*∗*^
The other patients who are in the clinic are all very athletic-looking	2.82 (0.84)	2.95 (0.81)	2.74 (0.68)	2.77 (1.02)	−0.14
The other patients who are in the clinic do not look very athletic	2.99 (0.82)	2.93 (0.79)	3.00 (0.78)	3.02 (0.91)	0.07
The other people who are in the clinic are very social (e.g., talk to each other a lot)	3.69 (1.10)	3.68 (0.96)	3.79 (1.06)	3.58 (1.28)	0.00
The other people who are in the clinic are not very social	2.06 (1.04)	2.05 (0.99)	1.96 (1.00)	2.19 (1.14)	0.04
The other people in the clinic are all people you would like to impress	2.44 (1.03)	2.45 (0.93)	2.68 (1.07)	2.16 (1.05)	−0.11
The other people in the clinic are all people you do not feel you need to impress	3.58 (1.00)	3.52 (0.98)	3.40 (1.01)	3.81 (0.98)	0.14
You are required to ear loose-fitting long pants and a long sleeve shirt (e.g., a track suit)	2.86 (1.14)	2.64 (1.04)	2.91 (1.06)	3.02 (1.30)	0.18^*∗*^
You are required to wear baggy shorts and a baggy t-shirt	3.05 (1.12)	2.55 (1.07)	3.23 (0.98)	3.37 (1.16)	0.23^*∗∗*^
You are required to wear a short, tight-fitting spandex top and bottoms	2.20 (1.07)	2.55 (1.00)	2.24 (1.14)	1.81 (0.98)	−0.31^*∗∗∗*^
Your physiotherapist is female	3.40 (0.84)	3.27 (0.62)	3.49 (0.91)	3.44 (0.96)	0.15
Your physiotherapist is male	2.79 (0.77)	2.89 (0.49)	2.79 (0.95)	2.70 (0.77)	−0.16

*Physical Environment*					
The physiotherapy clinic is one large open space	3.11 (1.18)	3.45 (1.07)	3.09 (1.18)	2.79 (1.23)	−0.23^*∗∗*^
The physiotherapy clinic consists of multiple treatment beds divided by a thin curtain	3.79 (1.00)	3.52 (1.07)	3.91 (0.89)	3.93 (1.01)	0.18^*∗*^
The physiotherapy clinic consists of many small, completely separate offices where patients are treated behind a closed door	2.28 (1.31)	2.14 (1.13)	2.19 (1.36)	2.53 (1.42)	0.09

*Note. *Low SPA = scores ranging from 10 to 20, moderate SPA = 21–28, and high SPA = 29–41 on the Social Physique Anxiety Scale (SPAS). The Pearson *r* values depict the correlation between preferences and SPA. SPA = Social Physique Anxiety. ^*∗*^*p* < 0.05, ^*∗∗*^*p* < 0.01, and ^*∗∗∗*^*p* < 0.00.

**Table 4:** Descriptive characteristics of injured participants.

Characteristic	Women (*n* = 62)	
	Median (range)	Correlation with SPA (Pearson *r*)
Age	40 (17–73)	0.04
Height (m)	1.65 (1.47–1.85)	0.07
Weight (kg)	65.77 (47.63–122.47)	0.30^*∗*^
BMI (kg/m^2^)	24.28 (18.65–42.43)	0.29^*∗*^
SPA	24.00 (11.00–45.00)	—

*Note*. BMI = Body Mass Index; SPA = Social Physique Anxiety. ^*∗*^*p* < 0.05.

**Table 5:** Ratings for SPIRQ items and correlation with SPA for injured participants.

Item	Women (*n* = 62)		
	Mean	SD	Pearson *r*
Weak	1.95	1.06	0.15
Anxious	1.98	1.19	0.27^*∗*^
Self-conscious	2.16	1.18	0.49^*∗∗*^
Relaxed	3.07	1.23	0.14
Uncoordinated	2.19	1.24	0.18
Unable to perform	2.15	1.16	0.03
Healthy	3.03	1.13	0.17
Fit	3.21	1.09	0.24
Unfocused	1.86	1.07	0.42^*∗∗*^
Energized	3.39	1.15	0.02
Difficult to work with	1.36	0.75	0.22
Tense	2.19	1.20	0.28^*∗*^
Strong	3.23	1.02	0.09
Composed	2.77	1.12	0.23
Unable to handle pressure	1.61	0.88	0.52^*∗∗*^
Frustrated	1.92	1.09	0.37^*∗∗*^
Unconcerned	1.95	1.06	−0.01
Coordinated	3.03	1.06	0.02
Unfit	2.37	1.19	0.43^*∗∗*^
In control of emotions	2.44	1.20	0.08
Focused	2.47	1.30	0.05
Tired	2.55	1.24	0.21
Confident	2.76	1.11	0.08
Dumb	1.53	0.90	0.21
Unhealthy	2.02	1.14	0.39^*∗∗*^
Easy to work with	2.16	1.28	0.15
Knowledgeable	2.63	1.06	0.18
Able to handle pressure	2.39	1.11	0.24
Unable to control emotions	1.87	1.09	0.17
Needy	1.73	1.06	0.11
Perform exercises well	2.82	1.15	−0.10
Independent	2.40	1.22	−0.05

*Note*. Pearson *r* values depict the correlation with SPA. SPA = Social Physique Anxiety. ^*∗*^*p* < 0.05, ^*∗∗*^*p* < 0.01.

**Table 6:** Environmental preferences for injured participants and correlations with SPA.

Item	Total Sample (*N* = 62)	Low SPA (*n* = 21)	Med SPA (*n* = 20)	High SPA (*n* = 21)	Pearson *r* (total sample)
	Mean (SD)				
*Social environment*					
The other patients in the clinic are female	3.23 (0.89)	3.10 (0.63)	2.90 (1.12)	3.67 (0.73)	0.28^*∗*^
The other patients in the clinic are male	2.16 (0.94)	2.29 (0.96)	2.10 (0.97)	2.10 (0.94)	−0.10
There are an equal number of males and females in the clinic	2.97 (0.85)	3.05 (0.74)	3.05 (0.95)	2.81 (0.87)	−0.19
The other patients who are in the clinic are all very athletic-looking	2.57 (0.88)	2.81 (0.680)	2.60 (0.88)	2.29 (1.01)	−0.24
The other patients who are in the clinic do not look very athletic	2.77 (0.84)	2.86 (0.48)	2.60 (0.88)	2.86 (1.06)	−0.09
The other people who are in the clinic are very social (e.g., talk to each other a lot)	2.78 (1.10)	2.79 (0.98)	2.85 (1.09)	2.71 (1.27)	−0.13
The other people who are in the clinic are not very social	2.20 (1.09)	2.26 (0.99)	2.11 (1.21)	2.23 (1.12)	0.03
The other people in the clinic are all people you would like to impress	2.09 (0.93)	2.15 (0.96)	2.26 (0.91)	1.87 (0.91)	−0.10
The other people in the clinic are all people you do not feel you need to impress	3.29 (1.14)	3.36 (0.91)	3.22 (1.24)	3.29 (1.29)	0.06
You are required to ear loose-fitting long pants and a long sleeve shirt (e.g., a track suit)	3.22 (0.94)	3.12 (1.04)	3.11 (1.02)	3.42 (0.72)	0.18
You are required to wear baggy shorts and a baggy t-shirt	2.98 (1.09)	2.95 (0.97)	3.05 (1.32)	2.95 (1.02)	−0.06
You are required to wear a short, tight-fitting spandex top and bottoms	1.38 (0.71)	1.70 (0.89)	1.02 (0.09)	1.41 (0.71)	−0.18
Your physiotherapist is female	3.13 (0.89)	3.20 (0.60)	3.01 (0.97)	3.17 (1.06)	0.02
Your physiotherapist is male	2.66 (0.75)	2.63 (0.72)	2.88 (0.72)	2.46 (0.78)	−0.07

*Physical Environment*					
The physiotherapy clinic is one large open space	2.39 (1.23)	3.00 (1.00)	2.05 (1.19)	2.10 (1.30)	−0.30^*∗*^
The physiotherapy clinic consists of multiple treatment beds divided by a thin curtain	3.36 (1.11)	3.19 (1.03)	3.50 (1.15)	3.38 (1.20)	0.03
The physiotherapy clinic consists of many small, completely separate offices where patients are treated behind a closed door	2.24 (1.38)	1.81 (1.21)	2.10 (1.41)	2.81 (1.37)	0.35^*∗∗*^

*Note. *Low SPA = scores ranging from 11 to 22, moderate SPA = 23–27, and high SPA = 28–45 on the Social Physique Anxiety Scale (SPAS). The Pearson *r *values depict the correlation between preferences and SPA. SPA = Social Physique Anxiety. ^*∗*^*p* < 0.05, ^*∗∗*^*p* < 0.01.
