# Supplementary material for: An Examination of Women's Self-Presentation, Social Physique Anxiety, and Setting Preferences during Injury Rehabilitation
Source: Rehabil Res Pract. 2017 Mar 12;2017:6126509. doi: 10.1155/2017/6126509 (PMC5366195; doi:10.1155/2017/6126509)

APPENDIX A

Self-Presentation in Injury Rehabilitation Questionnaire

Self-Presentation in Injury Rehabilitation Questionnaire (SPIRQ)

Imagine you are receiving the physiotherapy treatment for the injury described on the previous page. The clinic is one large open space similar to a gymnasium. In the middle of the space is an area where you will be required to perform your exercises (e.g., stretching and strengthening exercises). There are treatment beds around the perimeter of the exercise area where your hands-on (i.e., manual) treatment will be provided by your physiotherapist and where you will receive IFC and ice. There are many mirrors and windows in this space. Refer to the photos displayed at the front of the class.

Imagine that there are also many people in this clinic. There are people of all ages, all different body compositions (i.e., height and weight), and both males and females. The people consist of physiotherapists, athletic therapists, kinesiologists, receptionists, patients, parents/partners of patients, and researchers. Imagine that this is where you will be attending physiotherapy appointments.

Again, imagine that you are receiving the physiotherapy treatment for the injury described on the previous page, in the clinical setting described. Imagining how you think the *other people* in the clinic would perceive you respond to each of the following items. For example, if you think the other people would perceive you as weak, you would circle 4 or 5. However, you if think the other people would NOT perceive you as weak, you would circle 1 or 2.

I believe the other people in the clinic would perceive me as ….

**1.**	Weak	Not at all true	1	2	3	4	5	Completely true
**2.**	Anxious	Not at all true	1	2	3	4	5	Completely true
**3.**	Self-conscious	Not at all true	1	2	3	4	5	Completely true
**4.**	Relaxed	Not at all true	1	2	3	4	5	Completely true
**5.**	Uncoordinated	Not at all true	1	2	3	4	5	Completely true
**6.**	Unable to perform exercises	Not at all true	1	2	3	4	5	Completely true

**7.**	Healthy	Not at all true	1	2	3	4	5	Completely true
**8.**	Fit	Not at all true	1	2	3	4	5	Completely true
**9.**	Unfocused	Not at all true	1	2	3	4	5	Completely true
**10.**	Energized	Not at all true	1	2	3	4	5	Completely true
**11.**	Difficult to work with	Not at all true	1	2	3	4	5	Completely true
**12.**	Tense	Not at all true	1	2	3	4	5	Completely true
**13.**	Strong	Not at all true	1	2	3	4	5	Completely true
**14.**	Composed	Not at all true	1	2	3	4	5	Completely true
**15.**	Unable to handle pressures	Not at all true	1	2	3	4	5	Completely true
**16.**	Frustrated	Not at all true	1	2	3	4	5	Completely true
**17.**	Unconcerned	Not at all true	1	2	3	4	5	Completely true
**18.**	Coordinated	Not at all true	1	2	3	4	5	Completely true
**19.**	Unfit	Not at all true	1	2	3	4	5	Completely true
**20.**	In control of my emotions	Not at all true	1	2	3	4	5	Completely true

**21.**	Focused	Not at all true	1	2	3	4	5	Completely true
**22.**	Tired	Not at all true	1	2	3	4	5	Completely true
**23.**	Confident	Not at all true	1	2	3	4	5	Completely true
**24.**	Dumb	Not at all true	1	2	3	4	5	Completely true
**25.**	Unhealthy	Not at all true	1	2	3	4	5	Completely true
**26.**	Easy to work with	Not at all true	1	2	3	4	5	Completely true
**27.**	Knowledgeable	Not at all true	1	2	3	4	5	Completely true
**28.**	Able to handle pressures	Not at all true	1	2	3	4	5	Completely true
**29.**	Unable to control emotions	Not at all true	1	2	3	4	5	Completely true
**30.**	Needy	Not at all true	1	2	3	4	5	Completely true
**31.**	Perform exercises well	Not at all true	1	2	3	4	5	Completely true
**32.**	Independent	Not at all true	1	2	3	4	5	Completely true

APPENDIX B

Physiotherapy Clinic Photographs

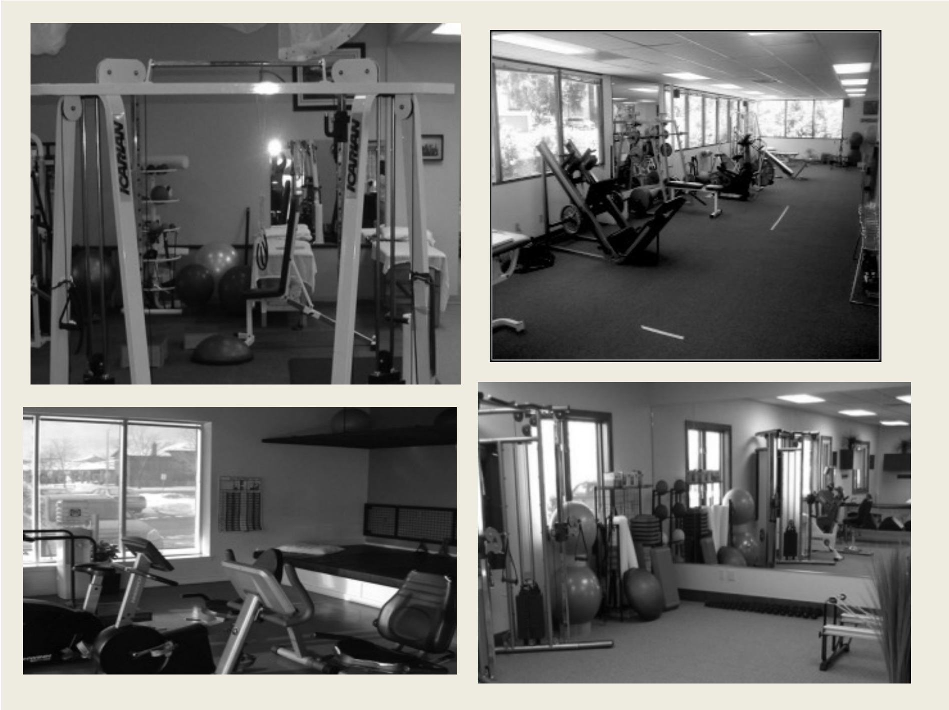
ICARIAN
ICARIAN

APPENDIX C

Injury Rehabilitation Social Environment Preferences Questionnaire

Injury Rehabilitation Social Environment Preferences Questionnaire

Imagine you are receiving the physiotherapy treatment for the injury described on the previous page. The clinic is one large open space similar to a gymnasium. In the middle of the space is an area where you will be required to perform your exercises (e.g., stretching and strengthening exercises). There are treatment beds around the perimeter of the exercise area where your hands-on (i.e., manual) treatment will be provided by your physiotherapist and where you will receive IFC and ice. There are many mirrors and windows in this space. Refer to the photos displayed at the front of the class.

Imagine that there are also many people in this clinic. There are people of all ages, all different body compositions (i.e., height and weight), and both males and females. The people consist of physiotherapists, athletic therapists, kinesiologists, receptionists, patients, parents/partners of patients, and researchers. Imagine that this is where you will be attending physiotherapy appointments.

Instructions: Again, imagining that you are in this physiotherapy clinic receiving treatment for the injury described, use the following 5-point scale to indicate the rating that best describes your preference for each statement.

1	2	3	4	5
**Not Preferred**		**No Preference**		**Completely Prefer**

1. The other patients in the clinic are the same sex as you. __________

2. The other patients in the clinic are of the opposite sex. ___________

3. There are an equal number of males and females in the clinic. __________

4. The other patients who are in the clinic are all very athletic-looking. _________

5. The other patients who are in the clinic do not look very athletic. _________

6. The other people who are in the clinic are very social (e.g., talk to each other a lot).____

7. The other people who are in the clinic are not very social. __________

8. The other people in the clinic are all people you would like to impress. __________

9. The other people in the clinic are people you do not feel you need to impress._______

10. You are required to wear loose-fitting long pants and a long sleeve shirt (e.g., a track suit).

____

11. You are required to wear baggy shorts and a baggy t-shirt. _________

12. You are required to wear a short, tight-fitting spandex top and bottoms.______

13. Your physiotherapist is the same sex as you. _________

14. Your physiotherapist is of the opposite sex as you. __________

APPENDIX D

Injury Rehabilitation Treatment Environment Preferences Questionnaire

Injury Rehabilitation Treatment Environment Preferences Questionnaire

Read the descriptions provided below and look at the photographs that accompany the descriptions. Imagine that this is the physiotherapy clinic where you will be attending physiotherapy appointments and receiving treatment for your current injury.

Instructions: Use the following 5-point scale to indicate the rating that best describes your preference for each physiotherapy clinic depicted by the written descriptions and accompanying photographs.

1	2	3	4	5
**Not Preferred**	**Slight Preference**	**No Preference**	**Much Preferred**	**Completely Preferred**

Continue to next page for photos and written descriptions.

1. The physiotherapy clinic is one large open space. There are treatment beds around the perimeter of the gym-like area where hands-on (i.e., manual) treatment is provided by the physiotherapist. Patients perform their exercises in the center of the open space. There are mirrors on most of the walls and there are windows that allow people outside of the clinic to see in. **Rating:________**

**1**	**2**	**3**	**4**	**5**
**Not Preferred**	**Slight Preference**	**No Preference**	**Much Preferred**	**Completely Preferred**

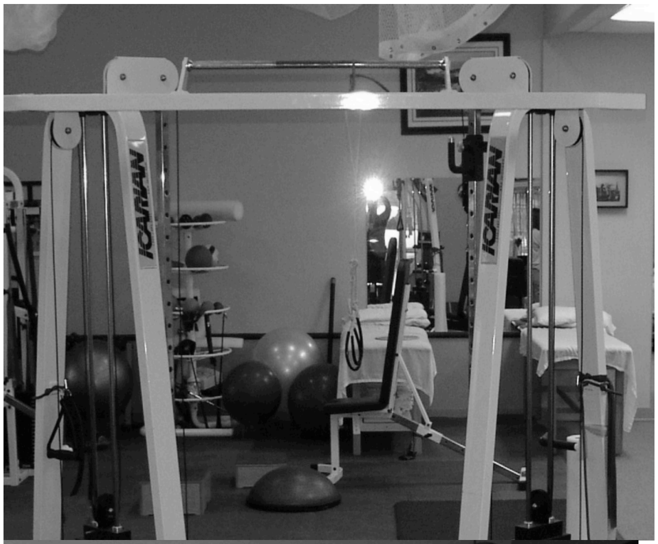

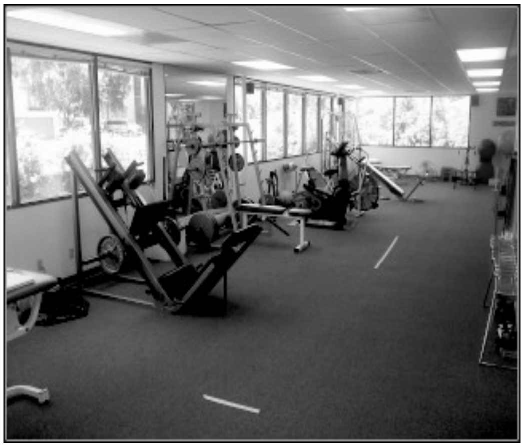
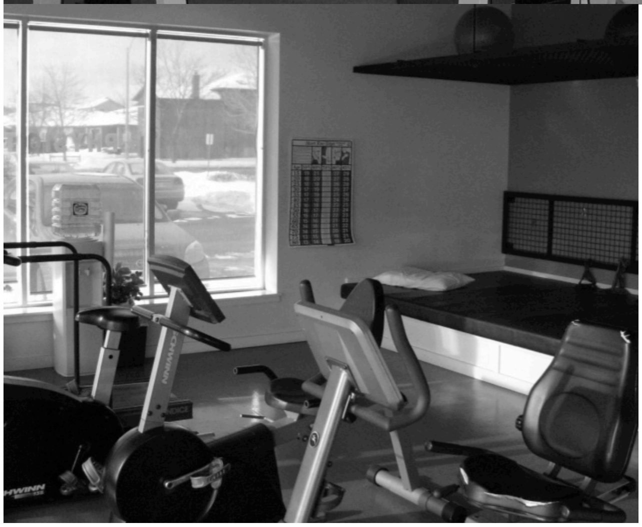
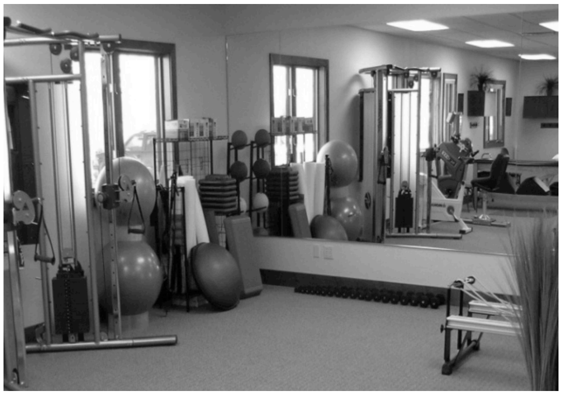

2. The physiotherapy clinic consists of multiple open spaces divided by partitions with treatment beds in one area which are each divided by a thin curtain. Hands-on (i.e., manual) treatment occurs on the beds behind the curtains. Others cannot see you, but are able to hear you. Exercises are completed in shared exercise areas. The exercise areas have mirrors on only one wall. There are few windows in the clinic and they are covered so that no one outside of the clinic can see in. **Rating:** _________

**1**	**2**	**3**	**4**	**5**
**Not Preferred**	**Slight Preference**	**No Preference**	**Much Preferred**	**Completely Preferred**

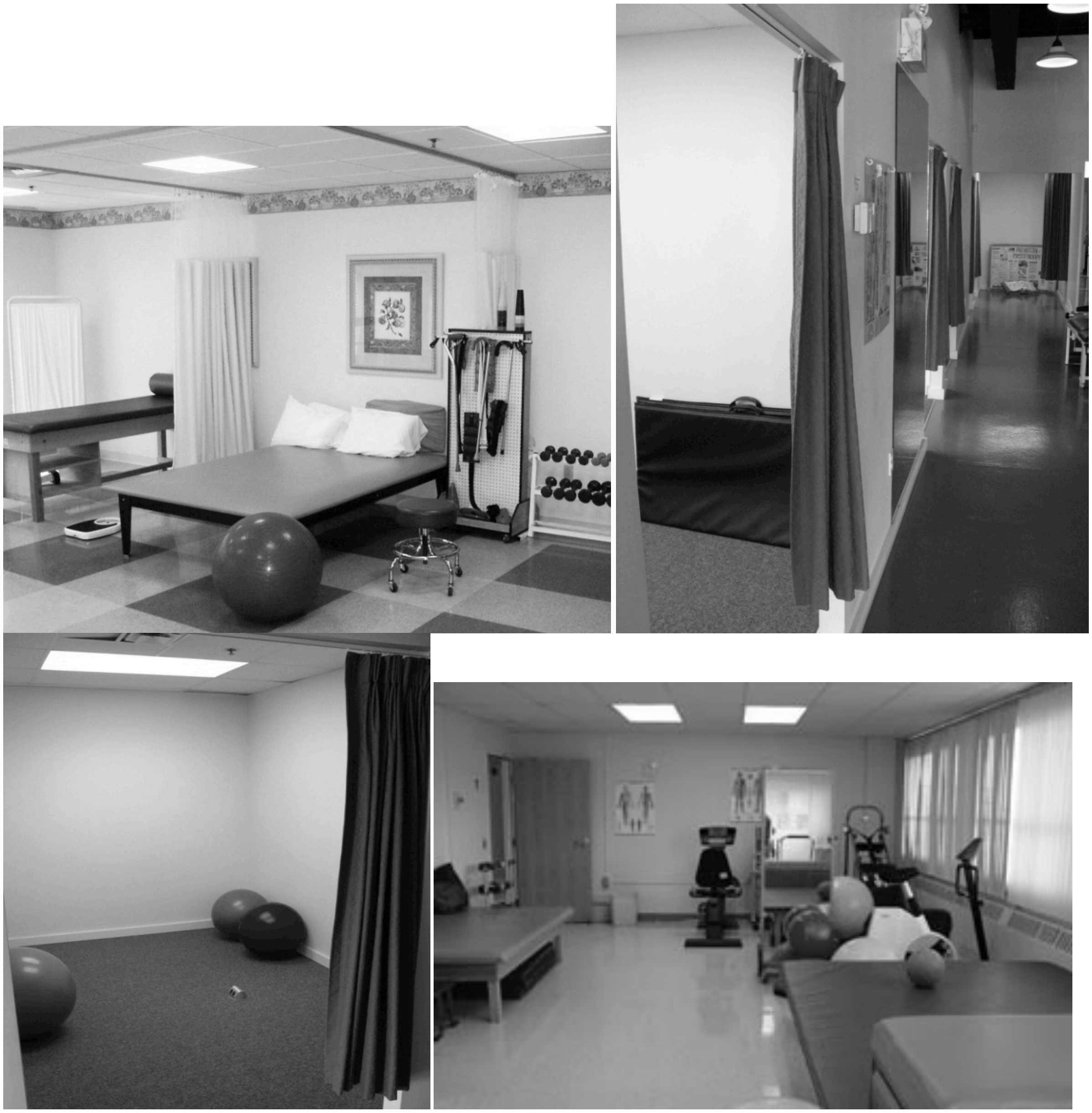

3. The physiotherapy clinic consists of many small, completely separate offices where patients are treated behind a closed door. Hands-on (i.e., manual) treatment occurs on the treatment beds within each room. Exercises are also completed in the individual rooms. There are no mirrors in the rooms. There are no windows in the rooms.
   Rating: _______

**1**	**2**	**3**	**4**	**5**
**Not Preferred**	**Slight Preference**	**No Preference**	**Much Preferred**	**Completely Preferred**

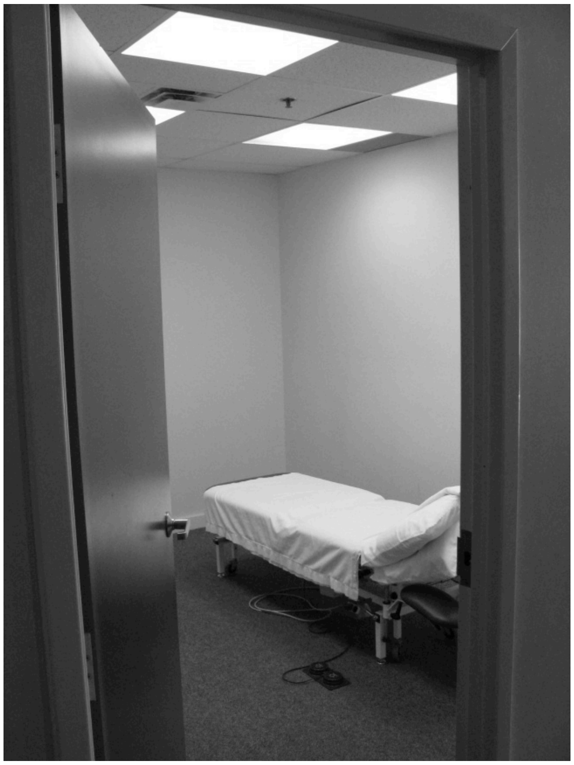

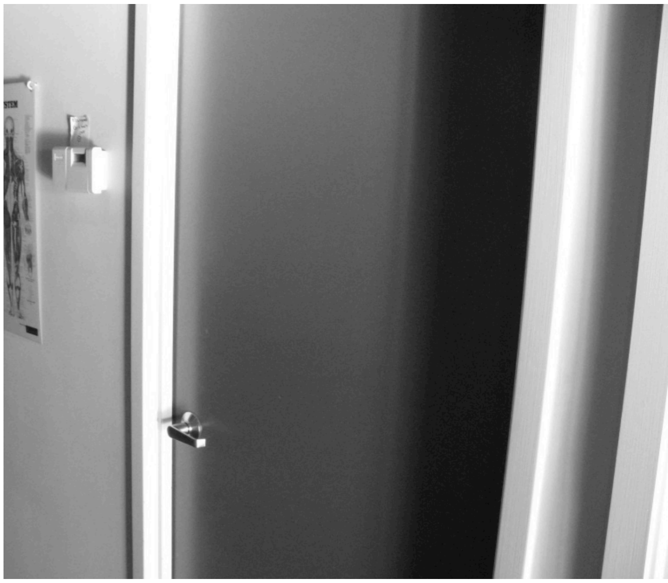

Supplement: Supplementary file 1 — The Supplementary Material contains the bespoke questionnaires created for the reported studies: the Self-Presentation in Injury Rehabilitation Questionnaire (SPIRQ), the Injury Rehabilitation Social Environment Preferences Questionnaire, and the Injury Rehabilitation Treatment Environment Preferences Questionnaire. [file 6126509.f1.pdf]
